# Comparing niraparib versus platinum-taxane doublet chemotherapy as neoadjuvant treatment in patients with newly diagnosed homologous recombination–deficient stage III/IV ovarian cancer: study protocol for cohort C of the open-label, phase 2, randomized controlled multicenter OPAL trial

**DOI:** 10.1186/s13063-024-08142-5

**Published:** 2024-05-04

**Authors:** Jimmy Belotte, Brunella Felicetti, Amanda J. Baines, Ahmed YoussefAgha, Luis Rojas-Espaillat, Ana Godoy Ortiz, Diane Provencher, Raúl Márquez Vázquez, Lucia González Cortijo, Xing Zeng

**Affiliations:** 1 GSK, 1000 Winter Street North #3300, Waltham, MA 02451 USA; 2 GSK, Stevenage, England UK; 3 GSK, Berkeley Heights, NJ USA; 4 Avera Medical Group Gynecologic Oncology, Sioux Falls, SD USA; 5 Hospital Regional Universitario and Hospital Virgen de La Victoria, Málaga, Spain; 6 Centre Hospitalier de l’Université de Montreal, Montréal, QC Canada; 7 MD Anderson Madrid, Madrid, Spain; 8 Hospital Quirón Madrid, Madrid, Spain; 9 McGill University Health Centre, Montréal, QC Canada

**Keywords:** Homologous recombination–deficient, Interval debulking surgery, Neoadjuvant chemotherapy, Niraparib, Ovarian cancer, Overall response rate, PARP inhibitor

## Abstract

**Background:**

Maintenance therapy with niraparib, a poly(ADP-ribose) polymerase inhibitor, has been shown to extend progression-free survival in patients with newly diagnosed advanced ovarian cancer who responded to first-line platinum-based chemotherapy, regardless of biomarker status. However, there are limited data on niraparib’s efficacy and safety in the neoadjuvant setting. The objective of Cohort C of the OPAL trial (OPAL-C) is to evaluate the efficacy, safety, and tolerability of neoadjuvant niraparib treatment compared with neoadjuvant platinum-taxane doublet chemotherapy in patients with newly diagnosed stage III/IV ovarian cancer with confirmed homologous recombination–deficient tumors.

**Methods:**

OPAL is an ongoing global, multicenter, randomized, open-label, phase 2 trial. In OPAL-C, patients will be randomized 1:1 to receive three 21-day cycles of either neoadjuvant niraparib or platinum-taxane doublet neoadjuvant chemotherapy per standard of care. Patients with a complete or partial response per Response Evaluation Criteria in Solid Tumors version 1.1 (RECIST v1.1) will then undergo interval debulking surgery; patients with stable disease may proceed to interval debulking surgery or alternative therapy at the investigator’s discretion. Patients with disease progression will exit the study treatment and proceed to alternative therapy at the investigator’s discretion. After interval debulking surgery, all patients will receive up to three 21-day cycles of platinum-taxane doublet chemotherapy followed by niraparib maintenance therapy for up to 36 months. Adult patients with newly diagnosed stage III/IV ovarian cancer eligible to receive neoadjuvant platinum-taxane doublet chemotherapy followed by interval debulking surgery may be enrolled. Patients must have tumors that are homologous recombination–deficient. The primary endpoint is the pre–interval debulking surgery unconfirmed overall response rate, defined as the investigator-assessed percentage of patients with unconfirmed complete or partial response on study treatment before interval debulking surgery per RECIST v1.1.

**Discussion:**

OPAL-C explores the use of niraparib in the neoadjuvant setting as an alternative to neoadjuvant platinum-taxane doublet chemotherapy to improve postsurgical residual disease outcomes for patients with ovarian cancer with homologous recombination–deficient tumors. Positive findings from this approach could significantly impact preoperative ovarian cancer therapy, particularly for patients who are ineligible for primary debulking surgery.

**Trial registration:**

ClinicalTrials.gov NCT03574779. Registered on February 28, 2022.

**Supplementary Information:**

The online version contains supplementary material available at 10.1186/s13063-024-08142-5.

## Introduction

Despite treatment advancements, the ovarian cancer mortality rate remains high. In 2022, there was an estimation of 26,500 ovarian cancer-related fatalities in the European Union and 4000 in the UK [1]. Projections indicate that there will be approximately 13,270 ovarian cancer-related fatalities in the United States in 2023 [2]. For patients with newly diagnosed advanced ovarian cancer, cytoreductive surgery and platinum-based chemotherapy are recommended [3]. Although the majority of patients respond favorably to initial treatment, most patients experience disease progression within 2 years and require additional treatment [4].

A clear unmet need to improve outcomes in patients with newly diagnosed advanced ovarian cancer exists due to high mortality rates [2], particularly those with advanced disease who cannot undergo primary debulking surgery and are treated with neoadjuvant chemotherapy followed by interval debulking surgery (IDS). Postsurgical residual disease negatively affects survival [5], and innovations in the neoadjuvant setting that can improve postsurgical residual disease status may significantly improve patient care.

International clinical trials such as SOLO-1 (NCT01844986) [6], PRIMA/ENGOT-OV26/GOG-3012 (NCT02655016) [7], PRIME (NCT03709316) [5], and ATHENA-Mono/GOG-3020/ENGOT-OV45 (NCT03522246) [8] have demonstrated benefits in progression-free survival when poly(ADP-ribose) polymerase (PARP) inhibitors are used for first-line maintenance treatment of patients with ovarian cancer with homologous recombination–deficient tumors. Niraparib and rucaparib demonstrated efficacy across biomarker subgroups but showed the greatest benefit in patients with *BRCA*-mutated or homologous recombination–deficient ovarian cancer [7, 8]. Niraparib, a potent and highly selective PARP-1 and PARP-2 inhibitor, is approved by both the United States Food and Drug Administration and the European Medicines Agency for first-line maintenance treatment of adult patients with advanced epithelial ovarian cancer with a complete or partial response to first-line platinum-based chemotherapy, regardless of biomarker status [9, 10].

The progression-free survival benefit of niraparib first-line maintenance therapy in patients with ovarian cancer [7] provides a strong rationale to evaluate the potential benefits of using niraparib in the neoadjuvant setting. In particular, given the continuum of niraparib benefit observed across biomarker subgroups, patients with homologous recombination–deficient tumors are the most likely candidates to benefit from neoadjuvant niraparib. Accordingly, cohort C of the OPAL trial (OPAL-C) was designed to evaluate the efficacy, safety, and tolerability of neoadjuvant niraparib treatment in patients with newly diagnosed advanced ovarian cancer with homologous recombination–deficient tumors.

## Methods

The reporting of this OPAL-C study protocol (version 2.0; Supplement C, Version 1.0, dated 13 September 2021) conforms to the Standard Protocol Items: Recommendations for Interventional Trials (SPIRIT) guidelines [11]. The order of the SPIRIT checklist items has been modified to group similar items together.

### Trial design

OPAL (NCT03574779) is a phase 1B/2 multicohort proof-of-concept umbrella study designed to evaluate the safety and efficacy of novel treatments and/or combinations of treatments in patients with ovarian cancer. Objectives are cohort-specific, and the design of each cohort is tailored to the patient population and treatment of interest. At the time of this publication, OPAL has 2 ongoing cohorts, cohort A and cohort C. Cohort A (OPAL-A) focuses on evaluating the efficacy and safety of the dostarlimab, bevacizumab, and niraparib combination in patients with recurrent platinum-resistant ovarian cancer who have received 1 to 2 prior lines of therapy. Notably, patients in cohort A must not have received prior treatment with PARP inhibitors. Cohort B was canceled before activation.

**Fig. 1:**
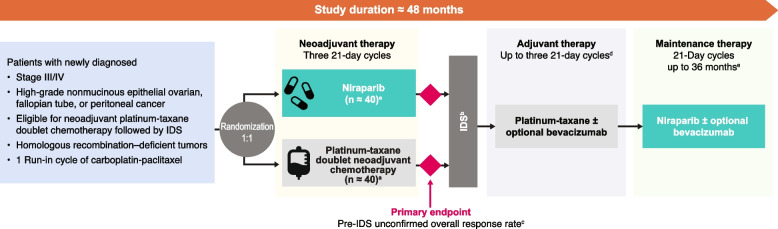
Study schematic. The study duration for each patient will be up to 48 months. ^a^An estimated sample size of 40 patients in each of the 2 arms (80 patients total) is needed for the half-width of a 2-sided 80% CI for the pre-IDS unconfirmed overall response rate difference of 12%. This estimate assumes that the pre-IDS unconfirmed overall response rate in the neoadjuvant niraparib arm is 65% and that the pre-IDS unconfirmed overall response rate difference between arms is 20%. ^b^Patients with a complete or partial response after neoadjuvant therapy per RECIST v1.1 go on to receive IDS. Patients with stable disease or disease progression may proceed to an alternative therapy at the investigator’s discretion. ^c^Defined as the percentage of patients with unconfirmed complete or partial response on study treatment before IDS as assessed by the investigator using the RECIST v1.1 criteria. ^d^Third cycle optional. ^e^Up to 36 months in the absence of progressive disease, death, unacceptable toxicity, or patient/physician decision to withdraw from the study. *CI*, confidence interval; *IDS*, interval debulking surgery; *RECIST v1.1*, Response Evaluation Criteria for Solid Tumors version 1.1

The global, multicenter, randomized, open-label, phase 2 OPAL-C cohort is focused on patients with newly diagnosed, advanced ovarian cancer (high-grade nonmucinous epithelial ovarian, fallopian tube, or peritoneal cancer, collectively referred to as ovarian cancer) with homologous recombination–deficient tumors who are eligible for neoadjuvant platinum-taxane doublet chemotherapy followed by IDS. Figure 1 and Table 1 show the OPAL-C schematic and study schedule, respectively. The estimated study duration for each patient will be up to 48 months.

**Table 1:**
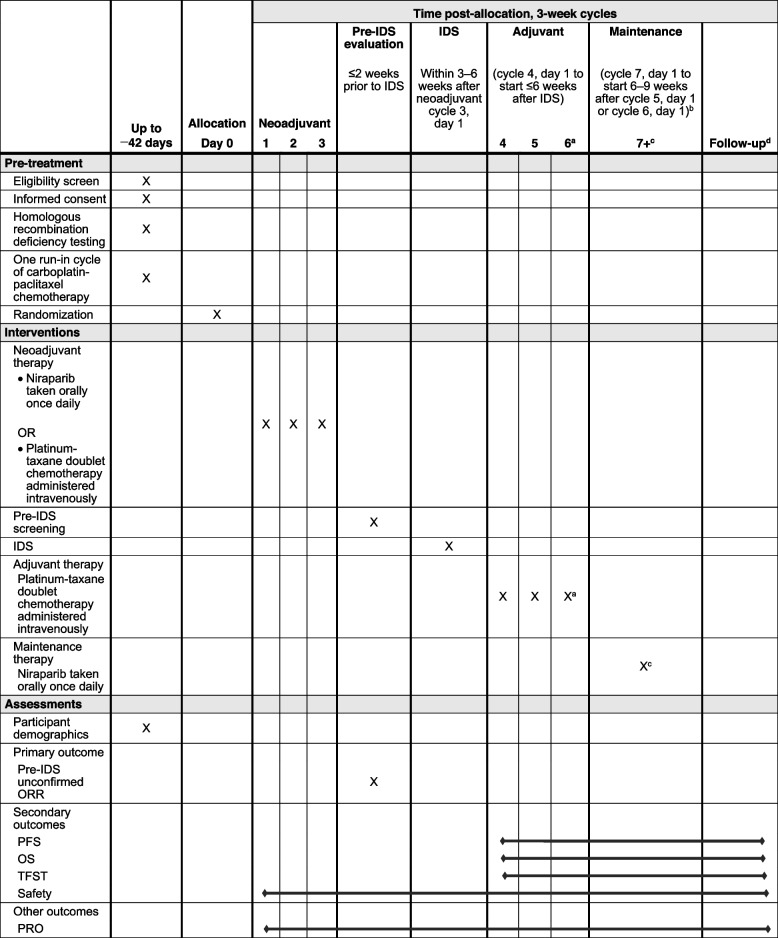
Schedule for enrolment, interventions, and assessments (SPIRIT figure)

*IDS* Interval debulking surgery, *ORR* Overall response rate, *OS* Overall survival, *PFS* Progression-free survival, *PRO* Patient-reported outcome, *TFST* Time to first subsequent treatment

^a^Third cycle of adjuvant therapy is optional

^b^Start of maintenance depends on whether patients receive the optional third cycle of adjuvant therapy

^c^Maintenance treatment in 3-week cycles for up to 36 months

^d^Includes safety and long-term follow-up

#### Participants

Table 2 shows the key eligibility criteria. Adult patients with stage III/IV ovarian cancer according to International Federation of Gynecology and Obstetrics staging guidelines and an Eastern Cooperative Oncology Group performance status ≤ 2 may enroll. All patients must have homologous recombination–deficient tumors as determined by central testing using the myChoice CDx PLUS assay (Myriad Genetics, Salt Lake City, UT, USA). Eligible patients with germline *BRCA1/2* deleterious or presumed deleterious mutations by an authorized test (e.g., BRACAnalysis CDx; Myriad Genetics) will need central homologous recombination deficiency testing using the myChoice CDx PLUS assay to confirm eligibility. Before study entry, patients must have completed 1 run-in cycle of carboplatin-paclitaxel. In addition to the exclusion criteria given in Table 2, patients who are immunocompromised or who have an autoimmune disease that has required systemic treatment in the last 2 years are ineligible.

**Table 2:** Key patient eligibility criteria

Key inclusion criteria	Key exclusion criteria
Must be female (aged ≥ 18 years) with measurable disease according to RECIST v1.1	Low-grade/grade 1 epithelial ovarian cancer or mucinous, germ or transitional cell, carcinosarcoma, and undifferentiated tumor
Stage III/IV ovarian, fallopian tube, or peritoneal cancer according to FIGO	Have contraindications to surgery
Have tumors that are homologous recombination deficient as determined by central homologous recombination deficiency testing using the myChoice CDx PLUS assay (Myriad Genetics, Salt Lake City, UT, USA)	Received prior treatment for high-grade nonmucinous epithelial ovarian, fallopian tube, or peritoneal cancer (e.g., prior surgery, immunotherapy, anticancer therapy [except for 1 run-in cycle of carboplatin-paclitaxel], or radiation therapy)
Prior completion of 1 run-in cycle of carboplatin-paclitaxel treatment and not experienced disease progression after this treatment. Completion is defined as receiving ≥ 50% of the prescribed dose of therapy within 5 weeks	Have bowel obstruction (clinical symptoms or computed tomography scan), subocclusive mesenteric disease, abdominal or gastrointestinal fistula, gastrointestinal perforation, or intra-abdominal abscess
Patient must not have known contraindication or uncontrolled hypersensitivity to platinum-taxane agents (e.g., carboplatin-paclitaxel) and no known pre-existing conditions that would preclude treatment with these agents	History or present diagnosis of myelodysplastic syndrome or acute myeloid leukemia
Absence of contraindication or uncontrolled hypersensitivity to niraparib	High risk of bleeding due to major injuries or major surgery within the past 28 days before the start of study treatment and/or a history of hemorrhagic stroke, transient ischemic attack, subarachnoid hemorrhage, or clinically significant hemorrhage within the past 3 months
Absence of symptomatic ascites or pleural effusions	Inability to swallow orally administered medication or have a gastrointestinal disorder likely to interfere with absorption of the study medication
Agree to complete patient-reported outcome and work productivity questionnaires for the entirety of the study	Received whole-blood transfusion within the 2 weeks before study entry

*FIGO* International Federation of Gynecology and Obstetrics, *RECIST v1.1* Response Evaluation Criteria in Solid Tumors version 1.1

#### Screening

All patients will undergo baseline assessments and central homologous recombination deficiency tumor tissue testing using the myChoice CDx PLUS assay during a 5-week prescreening period. All patients will receive one run-in cycle of carboplatin-paclitaxel per local standard of care because of the urgency of initiating therapy and the time required to receive central homologous recombination deficiency testing results. Patients with confirmed homologous recombination–deficient tumors who meet all eligibility criteria and have signed informed consent forms will be enrolled in the study after recovery from the run-in cycle of carboplatin-paclitaxel.

#### Randomization and blinding

Patients will be randomly assigned in a 1:1 ratio to receive three 21-day cycles of neoadjuvant platinum-taxane doublet chemotherapy (carboplatin-paclitaxel, arm 1) or neoadjuvant niraparib (arm 2) approximately 3 weeks after the initial run-in cycle of carboplatin-paclitaxel treatment. Randomization with a block size of four will be performed using an interactive voice/web response technology system. Randomization will be stratified based on *BRCA* mutational status. As an open-label trial, patients will not be blinded to study treatment. To mitigate the risk of introducing bias into the assessment of treatment effect, clinical and statistical team members involved in the study will remain blinded to treatment-sensitive data until database freeze. Before the database freeze, no data aggregation (efficacy, safety, or pharmacokinetics) by treatment arm will be performed, except for prespecified interim analyses. The study will be monitored by the Data Review Committee, and only the Data Review Committee will review the aggregate data from the prespecified interim analysis.

#### Neoadjuvant therapy

Patients randomized to neoadjuvant niraparib treatment will receive an individualized starting dose based on body weight and platelet count at the time of screening. Patients with a body weight < 77 kg or screening platelet count < 150,000/μL will receive a niraparib dose of 200 mg; patients with a body weight ≥ 77 kg and screening platelet count ≥ 150,000/μL will receive a niraparib dose of 300 mg. Niraparib will be taken orally once daily throughout each 21-day treatment cycle.

Patients randomized to neoadjuvant platinum-taxane doublet chemotherapy will receive carboplatin-paclitaxel without bevacizumab per local standard of care. On day 1 of each cycle, carboplatin will be administered at the investigator’s prescribed dose, intravenously over 60 min, followed by paclitaxel at a dose of 175 mg/m^2^, administered intravenously over 180 min. Before each carboplatin-paclitaxel administration, patients must have an absolute neutrophil count ≥ 1500/μL (≥ 1000/μL when granulocyte colony–stimulating factor will be administered), platelet count ≥ 100,000/μL, and hemoglobin levels ≥ 9 g/dL. If a patient is unable to tolerate carboplatin or paclitaxel, they will receive cisplatin or docetaxel, respectively, and remain in the treatment arm for analysis.

Neoadjuvant treatment may be discontinued because of toxicity, disease progression per Response Evaluation Criteria in Solid Tumors version 1.1 (RECIST v1.1), or investigator/medical monitor decision. Clinical progression based on signs, symptoms, physical examination results, or increased cancer antigen 125 (CA-125) (> 15% from lowest recorded value) will not necessitate discontinuation of neoadjuvant treatment but may trigger an additional scan per RECIST v1.1. If patients do not meet stable disease or progression criteria per RECIST v1.1, but the investigator believes it is in the patient’s best interest to discontinue neoadjuvant treatment, neoadjuvant therapy may be discontinued after consultation with the study’s medical monitor; in such an event, the additional scan performed for this evaluation will be used for the primary efficacy analysis. Patients who receive additional cycles of platinum-taxane doublet chemotherapy before IDS will be considered as having discontinued treatment and will undergo end-of-treatment procedures and provide scans for assessment per RECIST v1.1 after the 3 cycles of neoadjuvant therapy.

#### IDS and pre-IDS assessment

**Fig. 2:**
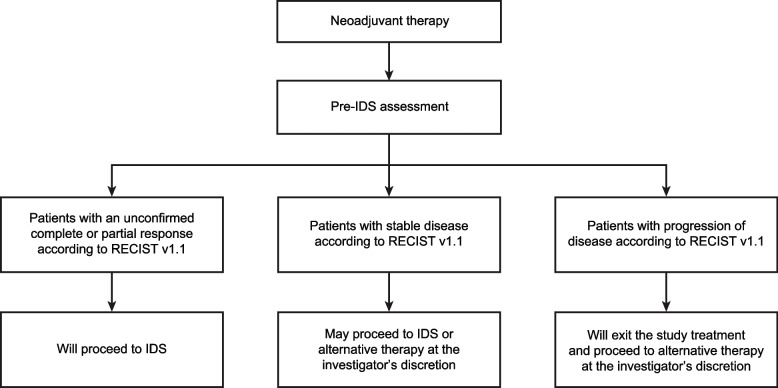
Criteria for selecting patients for IDS. *IDS*, interval debulking surgery; *RECIST v1.1*, Response Evaluation Criteria for Solid Tumors version 1.1

IDS will be performed 3 to 6 weeks after the final cycle of neoadjuvant therapy. Before surgery, patients will undergo a pre-IDS assessment. Figure 2 shows the criteria used for selecting patients for IDS. Patients with an unconfirmed complete or partial response according to RECIST v1.1 will undergo IDS by a qualified gynecologic/surgical oncologist aimed at achieving no visible residual disease. Patients with stable disease per RECIST v1.1 may proceed to IDS or an alternative therapy at the investigator’s discretion. Patients with disease progression will exit the study treatment and proceed to an alternative therapy at the investigator’s discretion. Surgical and postsurgical outcomes will be collected. Postsurgical safety will be assessed according to local standard of care for 30 days (± 7 days) after IDS and before adjuvant chemotherapy.

#### Adjuvant therapy

After a post-IDS recovery period of up to 6 weeks, all patients will receive up to three 21-day cycles of platinum-taxane doublet chemotherapy (and optional bevacizumab or biosimilar for patients deemed high risk by the investigator) regardless of neoadjuvant treatment arm assignment. The third treatment cycle will be optional. Platinum-taxane doublet chemotherapy will be administered identically to treatment arm 1 during neoadjuvant therapy. For patients deemed high risk by the investigator, 7.5 or 15 mg/kg of optional bevacizumab or biosimilar will be administered intravenously over 90 min. After adjuvant therapy and within 14 days before the first day of the first cycle of the maintenance treatment period, diagnostic imaging assessments per RECIST v1.1 will be performed.

#### Maintenance therapy

Patients without disease progression per RECIST v1.1 will start niraparib (and optional bevacizumab or biosimilar for patients deemed high risk by the investigator) maintenance treatment in 3-week cycles for up to 36 months. To enable patients to recover adequately from hematologic and nonhematologic toxicity, maintenance treatment can be delayed 6 to 9 weeks after the final adjuvant chemotherapy cycle.

Maintenance imaging assessments will be performed on the first day or within 14 days before the first day of the first maintenance cycle, then every 3 months (± 7 days) for 1 year after the first day of the first maintenance cycle, every 6 months (± 7 days) during the second year, and yearly thereafter until disease progression per RECIST v1.1, death, unacceptable toxicity, patient/physician withdrawal, or study completion. If a patient has increasing CA-125 levels or concerning symptoms, unscheduled computerized tomography or magnetic resonance imaging scans may be conducted per RECIST v1.1 criteria.

#### Treatment adjustment and discontinuation

Niraparib treatment can be interrupted because of adverse events for up to 14 and 21 days during neoadjuvant and maintenance treatment, respectively. Treatment will be interrupted for niraparib-related, nonhematologic, Common Terminology Criteria for Adverse Events grade ≥ 3 adverse events. Relation to study treatment will be determined by investigator assessment. Treatment interruption for hematologic adverse events will be determined based on prespecified blood counts. Additional file 1: Supplementary Table 1 shows the niraparib dose interruption and adjustment criteria for hematologic parameters.

If nonhematologic toxicity resolves to baseline or Common Terminology Criteria for Adverse Events grade ≤ 1 within 2 weeks for neoadjuvant therapy or within 3 weeks for maintenance treatment, patients may restart niraparib at a reduced dose if prophylaxis is not feasible. If the nonhematologic toxicity adverse event recurs at a similar or worse grade, treatment will be interrupted and a dose reduction made on resolution. Patients will discontinue niraparib treatment permanently if the toxicity requiring dose interruption does not resolve to baseline or Common Terminology Criteria for Adverse Events grade ≤ 1 or specified hematologic levels within the allowable period or if the patient has already had the maximum allowed 2 dose reductions.

Dose adjustments and supportive care for platinum-taxane doublet chemotherapy, bevacizumab, or biosimilar will follow guidelines specified in the prescribing information for each medication or local standard of care.

#### Concomitant medications

Any medication the patient takes other than the study treatment, including herbal and other nontraditional remedies, will be considered concomitant medications. For each patient in the study, a record will be kept of all concomitant medication that they are using or used, at screening and at each subsequent study visit. Permitted concomitant medications include contraceptives (only those prespecified in the protocol) and rescue medications for adverse events (as deemed necessary by the treating investigator according to local institutional practice and/or guidance in the appropriate prescribing information). During the treatment phase of the study, patients will be prohibited from receiving systemic anticancer or biological therapy, immunotherapy, chemotherapy (except for the chemotherapy treatments specified and approved for use in the study), hormonal therapy, radiation therapy, surgery (except for the prespecified IDS), live vaccines, and investigational agents other than niraparib and prophylactic cytokines (prohibited only in the first cycle of the study but allowed in subsequent cycles as per American Society of Clinical Oncology guidelines).

### Outcomes

The primary endpoint is pre-IDS unconfirmed overall response rate, defined as the investigator-assessed percentage of patients with unconfirmed complete or partial response on study treatment before IDS per RECIST v1.1. Secondary endpoints were the incidence of patients with CA-125 progression per Gynecological Cancer InterGroup CA-125 response criteria, progression-free survival (time from randomization to earliest date of disease progression per RECIST v1.1 by investigator assessment or death by any cause), and progression-free survival rate at 12, 18, and 24 months (proportion of participants without documented disease progression per RECIST v1.1 per investigator assessment or death within 12, 18, or 24 months after randomization); overall survival (time from randomization to the date of death by any cause); and time to first subsequent treatment (date of treatment randomization to the date of first subsequent anticancer therapy or death). Patient-reported outcomes will also be assessed using the Patient Reported Outcomes version of the Common Terminology Criteria for Adverse Events (PRO-CTCAE), Functional Assessment of Cancer Therapy- General Population (FACT-GP5), European Organization for Research and Treatment of Cancer (EORTC) IL136, and the EORTC IL137. For the PRO-CTCAE, selected items (taste changes, nausea, vomiting, abdominal pain, bloating, constipation, diarrhea, concentration, memory, muscle pain, joint pain, fatigue, anxiety, neuropathy, and sadness) will be assessed weekly during neoadjuvant chemotherapy and at end of treatment. FACT-GP5, EORTC IL136, and EORTC IL137 assessments will be collected weekly during the neoadjuvant treatment period, every 3 cycles for the first 13 to 14 months, and then every 3 months thereafter during niraparib maintenance treatment. Exploratory endpoints include pathological complete response rate, identification of potential disease or treatment-related biomarkers, and evaluation of surgical and postsurgical outcomes. A neoadjuvant treatment modality response score may be investigated post hoc if needed.

### Safety

Known risks associated with niraparib treatment include hematological adverse events, such as thrombocytopenia, and the development of myelodysplastic syndrome or acute myeloid leukemia. Both niraparib and bevacizumab have also been associated with an increased risk of hypertension. Within OPAL-C, safety will be evaluated based on the incidence and severity of treatment-emergent adverse events, serious adverse events, treatment discontinuations or dose delays or reductions due to adverse events, predefined adverse events of special interest (i.e., myelodysplastic syndrome, acute myeloid leukemia, and secondary cancer), changes in Eastern Cooperative Oncology Group performance status, changes in clinical laboratory results (hematology and chemistry), vital sign measurements, observations during symptom-directed physical examination, and use of concomitant medications. Events may be reported spontaneously or solicited through patient questioning and/or review of clinical data. All adverse events, regardless of causality, will be collected and recorded for each participant; events will be coded using the current version of the Medical Dictionary for Regulatory Activities coding system.

### Statistical methods

#### Sample size

A total of 84 patients are planned for randomization, such that an estimated sample size of 40 patients per treatment arm is evaluable for pre-IDS unconfirmed overall response rate. The sample size estimation was calculated using EAST software, version 6.5 (Cytel, Waltham, MA, USA). With 40 patients in each treatment arm, the half width of a 2-sided 80% confidence interval (CI) for the pre-IDS unconfirmed overall response rate difference was 12%. This estimate assumes that the pre-IDS unconfirmed overall response rate in the platinum-taxane neoadjuvant treatment arm is 65% and that the difference between treatment arms is 20%. Estimates for expected effect size and overall response rate were based on findings from the niraparib literature as well as internal GSK data.

#### Interim analysis

A single planned interim analysis for futility using the pre-IDS unconfirmed overall response rate will be performed when at least 30 patients (approximately 15 patients per treatment arm) have pre-IDS radiographic data to determine their responses. Per the prespecified boundaries, the study will be allowed to continue unchanged if there is a greater than 30% predictive probability that the lower limit of the 80% CI of the pre-IDS overall response rate difference is greater than 0 at the study’s end. The interim analysis will be performed by a contract research organization, with results reviewed by the independent Data Review Committee. Enrollment may be paused during the interim analysis.

#### Analysis populations

This study will involve 6 analysis populations. The intent-to-treat population will include all randomized patients, while the safety population will consist of all patients who received at least one dose of the study treatment. The efficacy population will include all patients in the safety population with measurable disease at baseline tumor assessment, defined as the presence of at least one target lesion. The response-evaluable population will include all patients in the efficacy population with at least one evaluable postbaseline tumor assessment. The biomarker population will comprise all patients with at least one follow-up tumor assessment and a tumor or blood sample. Lastly, the pharmacokinetic population will include all patients who received niraparib and have at least one measurable niraparib concentration.

#### Outcome assessment

The primary endpoint of pre-IDS unconfirmed overall response rate will be analyzed in the response-evaluable population. The 2-sided 80% CI for the pre-IDS unconfirmed overall response rate difference between the 2 treatment arms will be evaluated. For the secondary endpoint of CA-125 progression per Gynecological Cancer InterGroup CA-125 response criteria, incidences for each study period (i.e., neoadjuvant treatment, adjuvant treatment, and maintenance treatment) will be compared between treatment arms with the 95% CI calculated using the stratified Miettinen and Nurminen’s method (stratification factor, *BRCA* mutation status). Kaplan–Meier plots of the survival distribution function will be generated and the median, 25th, and 75th percentiles and corresponding 95% CI will be estimated for each treatment arm. Progression-free survival rates at 12, 18, and 24 will be compared across treatment arms. Progression-free survival, overall survival, and time to first subsequent treatment, the distribution for each treatment arm will be estimated using the Kaplan–Meier method and will be compared between the 2 treatment arms using log-rank test stratified by the stratification factor used for randomization (i.e., *BRCA* mutational status). Hazard ratios and corresponding 90% CI will be estimated from the Cox proportional hazard model.

PRO-CTCAE scores for each item attribute (frequency, severity, and/or interference) will be summarized by visit. The frequency and proportions of participants in each response category for the FACT-GP5 at each time point will be evaluated. For the EORTC IL136 and EORTC IL137, summary statistics for observed values and changes from baseline will be calculated for scales and items by treatment group at each time point.

Demographic and baseline clinical characteristics will be summarized: number and percentage for categorical variables and the number of patients, mean, standard deviation, median, minimum, and maximum for continuous variables.

## Trial information

### Registration

The trial is registered on ClinicalTrials.gov as NCT03574779 (https://clinicaltrials.gov/study/NCT03574779). All items from the World Health Organization Trial Registration Data Set (version 1.3.1) are present within the ClinicalTrials.gov record or within the approved protocol. Study-related inquiries can be addressed to GSKClinicalSupportHD@gsk.com.

### Trial status

OPAL-C is being performed by GSK (Durham, NC, USA) and is currently enrolling, with study sites in Canada, Spain, and the United States. Recruitment began on 14 April 2022, and the estimated date for completing accrual is 29 March 2024; the estimated date for presentation of results is September 2024. The protocol is version 2.0; Supplement C, Version 1.0, dated 13 September 2021.

### Roles and responsibilities

The sponsor, GSK, is coordinating the trial and is involved in overall study activities including study design creation; the collection, management, analysis and interpretation of data; and data dissemination.

The principal investigator(s) at each study site will be responsible for providing the institutional review board (IRB) or independent ethics committee (IEC) with progress reports and informing them of any serious adverse drug reactions and amendments to the protocol, in accordance with local requirements.

The principal investigator(s) at each study site will also ensure that the patient is given full and adequate oral and written information about the nature, purpose, possible risk, and benefit of the study. Informed consent will be administered by the principal investigator, study physician, or designated personnel, in accordance with each site’s standard operating procedure. Patients will be approached to join the study before treatment plan finalization, typically at the time of diagnosis. Overall, patients will be given the opportunity to ask questions, allowed time to consider the information provided, and notified that they are free to discontinue from the study at any time. The patient’s signed and dated informed consent will be obtained before conducting the study.

To ensure adequate participant enrollment, the local and central study teams will meet during the study’s initiation phase to develop enrollment projections for each study site and country. During the enrollment period, the teams will convene to identify any new challenges or obstacles and to determine the corresponding corrective actions.

To ensure adherence to the specified interventions in this trial, a central and on-site monitoring plan has been developed. The risks and trends identified by the central monitors will be reviewed quarterly to identify and implement the appropriate mitigation or corrective actions. On-site monitors will review patient charts to ensure adherence with all study procedures and report all major nonadherence issues and protocol deviations for the study team’s review. The study team will also hold regular medical data and protocol deviation reviews to ensure the trial remains adherent with the allocated interventions.

Because genetic variation may impact a patient’s response to study treatment, a blood sample for DNA isolation will be collected on day 1 of the first neoadjuvant treatment cycle from patients who have consented to participate in the genetics analysis component of the study. Participation in the genetic research will be optional, and patients who do not wish to participate in the genetic research may still participate in the study. Patients may withdraw their consent and have their specimens and all derivates destroyed.

### Data collection and management

A RAVE electronic data capture database will be used for data collection and storage. Data will be entered directly into the database (no paper forms) by trained and authorized research staff or study investigators. Patient records and the data generated by the study will be stored in a secure lockable location, and access to electronic data will be protected through a password-protected web interface and restricted to authorized users only. The data collected will be coded using a unique study number, and information collected on the electronic case report form will be coded using a unique subject number. After collecting all patient data, a member of the data management team and a medical staff member, both blinded to the randomization, will independently conduct a thorough manual review of the data to ensure accuracy and completeness. All patients records and the data generated will be confidential, in line with the recommendations of and in accordance with local requirements. Each principal investigator at every study site is responsible for ensuring the confidentiality and security of patient data. Any information that may identify a participant will be excluded from data presented in the public domain.

To safeguard the interest and safety of the patients in this trial, a GSK Data Review Committee, independent of the GSK study team directly involved in the conduct of the trial, will be used to review incoming data in order to monitor efficacy and emerging safety signals. The panel may recommend modifications to the design of the protocol or discontinuation of the trial, if necessary.

Authorized representatives of the sponsor, a regulatory authority, an IRB, or an IEC may visit the investigational site to perform audits or inspections, including source data verifications. The purpose of a sponsor audit or inspection will be to systematically and independently examine all study-related activities and documents to determine whether these activities were conducted, and data were recorded, analyzed, and accurately reported according to the protocol, Good Clinical Practice guidelines of the International Conference on Harmonization, and any applicable regulatory requirements.

### Biological samples

Biological samples, including required tumor tissue samples, will be given identification codes and stored securely for up to 15 years after the end of the study. Additional testing and analysis of biological samples will be subjected to the provisions agreed to under each patient’s signed informed consent form, and patients can request that their samples be destroyed at any time.

### Post-trial care

In the unfortunate event that a patient sustains an injury as a result of using the study drug or trial procedures, GSK will pay for the patient’s reasonable and necessary medical care.

### Dissemination plan

Authorship for future trial publications will be determined in accordance with established publication best practices, which include Good Publication Practices 2022 [12] and the International Committee of Medical Journal Editors guidelines [13]. If a large number of publications are planned, a dedicated Publication Committee may be established. Additionally, results of the OPAL-C trial will be reported according to the recommendations of the Consolidated Standards of Reporting Trials (CONSORT) statement, and publication in international open-access peer-reviewed journals with/without the assistance of professional writers is intended. Lastly, the final report of the trial will be reviewed by all trial sites.

## Discussion

Patients with newly diagnosed advanced ovarian cancer who are ineligible for primary debulking surgery are treated with neoadjuvant chemotherapy followed by IDS [5]. Results of a per-protocol pooled analysis of long-term follow-up data from the EORTC 55971 study [14] and the CHORUS research study [15] revealed that primary debulking surgery and IDS result in comparable overall survival, with neoadjuvant chemotherapy providing greater survival in patients with stage IV cancer [16]. Regardless of the timing and type of debulking surgery, the extent of residual disease after surgery is an important predictor of patient outcomes [14]. Therefore, there is a high unmet need to improve neoadjuvant therapy to provide surgeons the greatest possibility of achieving complete resection and patients the best possible prognosis. In addition to OPAL-C, use of the PARP inhibitors olaparib and niraparib as neoadjuvant treatment before debulking surgery is being evaluated in 2 other studies to improve patient outcomes [17, 18].

The aim of OPAL-C is to improve postsurgical residual disease outcomes and to minimize treatment-related adverse effects by using niraparib compared with platinum-taxane doublet chemotherapy in the neoadjuvant setting in patients with ovarian cancer with homologous recombination–deficient tumors. Positive findings from OPAL-C could significantly impact ovarian cancer treatment, particularly for patients who are ineligible for primary debulking surgery, and may lead to patients receiving less aggressive therapy (i.e., a chemotherapy-free PARP inhibitor) before surgery. Furthermore, efficacy data from niraparib usage in a neoadjuvant setting could be considered a surrogate for niraparib use in an adjuvant setting for patients with advanced ovarian cancer with homologous recombination–deficient tumors who have undergone debulking surgery. Combining neoadjuvant treatment and biopsies can yield valuable insights into the tumor microenvironment, enabling the identification of resistance mechanisms and response biomarkers. Additionally, pathological complete response evaluation might also act as a surrogate endpoint for ovarian cancer therapies. The expected favorable patient outcomes from OPAL-C will help establish rationale for future treatment combinations aimed at improving the prognosis of patients with newly diagnosed advanced ovarian cancer.

### Supplementary Information

**Additional file 1: Supplementary Table 1.** Niraparib dose adjustments for hematologic toxicity.

**Additional file 2.**

## Data Availability

The data generated by the current study protocol can be accessed by all listed authors. After completion of the trial, the data obtained by the trial will be summarized and analyzed according to this protocol and hereafter published in a peer reviewed journal to be assessable by any healthcare professional, participant, or the public. Raw data or additional data can be requested on demand upon reasonable request. Details regarding access to the statistical analysis plan (SAP) will be provided alongside the primary manuscript, which will present the study findings.

## Abbreviations

CA-125: Cancer antigen 125
CI: Confidence interval
EORTC IL136: European Organization for Research and Treatment of Cancer Item Library 136
EORTC IL137: European Organization for Research and Treatment of Cancer Item Library 137
FACT-GP5: Functional Assessment of Cancer Therapy-Item GP5
IDS: Interval debulking surgery
IEC: Independent ethics committee
IRB: Institutional review board
OPAL-C: OPAL cohort C
PARP: Poly(ADP-ribose) polymerase
PRO-CTCAE: Patient-Reported Outcomes version of the Common Terminology Criteria for Adverse Events
RECIST v1.1: Response Evaluation Criteria in Solid Tumors version 1.1
SPIRIT: Standard Protocol Items: Recommendations for Interventional Trials
