# Supplementary material for: Comparing niraparib versus platinum-taxane doublet chemotherapy as neoadjuvant treatment in patients with newly diagnosed homologous recombination–deficient stage III/IV ovarian cancer: study protocol for cohort C of the open-label, phase 2, randomized controlled multicenter OPAL trial
Source: Trials. 2024 May 4;25:301. doi: 10.1186/s13063-024-08142-5 (PMC11069300; doi:10.1186/s13063-024-08142-5)
Supplement: Supplementary file 1 — Additional file 1: Supplementary Table 1. Niraparib dose adjustments for hematologic toxicity. [file 13063_2024_8142_MOESM1_ESM.pdf]

Supplemental Materials

Supplementary materials have been provided by the authors to give readers additional information about their work.

Supplement to Comparing niraparib versus platinum-taxane doublet chemotherapy as neoadjuvant treatment in patients with newly diagnosed homologous recombination–deficient stage III/IV ovarian cancer: study protocol for cohort C of the open-label, phase 2, randomized controlled multicenter OPAL trial.

Supplementary Table 1 Niraparib dose adjustments for hematologic toxicity

Event[a]	Intervention
Platelet count <100,000/µL	First occurrence:<br>• Withhold niraparib for a maximum of 14 (neoadjuvant) or 21 (maintenance) days and monitor blood counts weekly until platelet counts return to ≥100,000/µL<br>• Resume niraparib at same or reduced dose[b]<br>• If platelet count <75,000/µL, resume niraparib at a reduced dose
	Second occurrence:<br>• Withhold niraparib for a maximum of 14 (neoadjuvant) or 21 (maintenance) days and monitor blood counts weekly until platelet counts return to ≥100,000/µL<br>• Resume niraparib at a reduced dose[b]<br>• Discontinue niraparib if the platelet count has not returned to acceptable levels within 14 (neoadjuvant) or 21 days (maintenance) of the dose interruption period or if the patient has already undergone dose reduction to 100 mg once daily
Neutrophil count[c] <1000/µL	Withhold niraparib for a maximum of 14 (neoadjuvant) or 21 (maintenance) days and monitor blood counts until neutrophil counts return to ≥1500/µL<br>Resume niraparib at a reduced dose[b]<br>Discontinue niraparib if neutrophil level has not returned to acceptable levels within 14 (neoadjuvant) or 21 days (maintenance) of the dose interruption period or if the patient has already undergone dose reduction to 100 mg once daily<br>Prophylactic cytokines administration[c]
Hemoglobin ≤8 g/dL	• Withhold niraparib for a maximum of 14 (neoadjuvant) or 21 (maintenance) days and monitor blood counts until hemoglobin returns to ≥9 g/dL<br>• Resume niraparib at a reduced dose[b]<br>• Discontinue niraparib if hemoglobin has not returned to acceptable levels within 14 (neoadjuvant) or 21 days (maintenance) of the dose interruption period, or if the patient has already undergone dose reduction to 100 mg once daily
Hematologic adverse reaction requiring transfusion	• For patients with platelet count ≤10,000/µL, platelet transfusion should be considered. If there are other risk factors for bleeding such as co-administration of anticoagulation or antiplatelet drugs, consider interrupting these drugs or transfusion at a higher platelet count.<br>• Red blood cell transfusion may be given at the discretion of the investigator<br>• Resume niraparib at a reduced dose[b]
Confirmed diagnosis of acute myeloid leukemia or myelodysplastic syndrome	Permanently discontinue niraparib

[a]Complete blood counts to be monitored weekly for the first cycle and once every treatment cycle thereafter.

[b]Niraparib dose must not be decreased to below 100 mg daily.

[c]Prophylactic cytokines (ie, granulocyte colony–stimulating factor) should not be administered in the first cycle of the study but may be administered in subsequent cycles according to current American Society of Clinical Oncology guidelines.
