# Supplementary material for: Comparing niraparib versus platinum-taxane doublet chemotherapy as neoadjuvant treatment in patients with newly diagnosed homologous recombination–deficient stage III/IV ovarian cancer: study protocol for cohort C of the open-label, phase 2, randomized controlled multicenter OPAL trial
Source: Trials. 2024 May 4;25:301. doi: 10.1186/s13063-024-08142-5 (PMC11069300; doi:10.1186/s13063-024-08142-5)
Supplement: Supplementary file 2 — Additional file 2. [file 13063_2024_8142_MOESM2_ESM.pdf]

SPIRIT Checklist for *Trials*

Complete this checklist by entering the page and line numbers where each of the items listed below can be found in your manuscript.

Your manuscript may not currently address all the items on the checklist. Please modify your text to include the missing information. If you are certain that an item does not apply, please state "n/a" and provide a short explanation. **Leaving an item blank or stating "n/a" without an explanation will lead to your manuscript being returned before review.**

Upload your completed checklist as an additional file when you submit to *Trials*. You must reference this additional file in the main text of your protocol submission. The completed SPIRIT figure must be included within the main body of the protocol text and can be downloaded here: http://www.spirit-statement.org/schedule-of-enrolment-interventions-and-assessments/

In your methods section, please state that you used the SPIRIT reporting guidelines, and cite them as:

Chan A-W, Tetzlaff JM, Gøtzsche PC, Altman DG, Mann H, Berlin J, Dickersin K, Hróbjartsson A, Schulz KF, Parulekar WR, Krleža-Jerić K, Laupacis A, Moher D. SPIRIT 2013 Explanation and Elaboration: Guidance for protocols of clinical trials. BMJ. 2013;346:e7586

		Reporting Item	Page and Line Number	Reason if not applicable
**Administrative information**				
Title	#1	Descriptive title identifying the study design, population, interventions, and, if applicable, trial acronym	Manuscript version: "OPALC Prot Ms_Trials_Rev1_30Jan2024_clean"<br>Page 1<br>Lines 1-4	
Trial registration	#2a	Trial identifier and registry name. If not yet registered, name of intended registry	Page 5, Line 69<br>Page 17, Lines 336-338	
Trial registration: data set	#2b	All items from the World Health Organization Trial Registration Data Set	17<br>Lines 336-338	

Protocol version	#3	Date and version identifier	7<br><br>Lines 106-107	
Funding	#4	Sources and types of financial, material, and other support	24<br><br>Line 480	
Roles and responsibilities: contributorship	#5a	Names, affiliations, and roles of protocol contributors	24-25<br><br>Line 481-500	
Roles and responsibilities: sponsor contact information	#5b	Name and contact information for the trial sponsor	Page 17, Line 345<br><br>Page 17, Line 338	
Roles and responsibilities: sponsor and funder	#5c	Role of study sponsor and funders, if any, in study design; collection, management, analysis, and interpretation of data; writing of the report; and the decision to submit the report for publication, including whether they will have ultimate authority over any of these activities	17<br><br>Lines 345-347	
Roles and responsibilities: committees	#5d	Composition, roles, and responsibilities of the coordinating centre, steering committee, endpoint adjudication committee, data management team, and other individuals or groups overseeing the trial, if applicable (see Item 21a for data monitoring committee)	17-18<br><br>Lines 347-375	
**Introduction**			6	

			Lines 74-104	
Background and rationale	#6a	Description of research question and justification for undertaking the trial, including summary of relevant studies (published and unpublished) examining benefits and harms for each intervention	6-7<br><br>Lines 87-102	
Background and rationale: choice of comparators	#6b	Explanation for choice of comparators	6<br><br>Lines 93-102	
Objectives	#7	Specific objectives or hypotheses	7<br><br>Lines 102-104	
Trial design	#8	Description of trial design including type of trial (eg, parallel group, crossover, factorial, single group), allocation ratio, and framework (eg, superiority, equivalence, non-inferiority, exploratory)	7-8<br><br>Lines 111-124	
**Methods: Participants, interventions, and outcomes**				
Study setting	#9	Description of study settings (eg, community clinic, academic hospital) and list of countries where data will be collected. Reference to where list of study sites can be obtained	17<br><br>Lines 340-341	
Eligibility criteria	#10	Inclusion and exclusion criteria for participants. If applicable, eligibility criteria for study centres and individuals who will	8<br><br>Lines 126-135<br><br>Also in Table 2, page 29	

		perform the interventions (eg, surgeons, psychotherapists)		
Interventions: description	#11a	Interventions for each group with sufficient detail to allow replication, including how and when they will be administered	9-11<br><br>Lines 157-213	
Interventions: modifications	#11b	Criteria for discontinuing or modifying allocated interventions for a given trial participant (eg, drug dose change in response to harms, participant request, or improving / worsening disease)	12<br><br>Lines 215-231<br><br>Also found in Supplementary Table 1	
Interventions: adherance	#11c	Strategies to improve adherence to intervention protocols, and any procedures for monitoring adherence (eg, drug tablet return; laboratory tests)	18<br><br>Lines 364-369	
Interventions: concomitant care	#11d	Relevant concomitant care and interventions that are permitted or prohibited during the trial	12-13<br><br>Lines 233-244	
Outcomes	#12	Primary, secondary, and other outcomes, including the specific measurement variable (eg, systolic blood pressure), analysis metric (eg, change from baseline, final value, time to event), method of aggregation (eg, median, proportion), and time point for each outcome. Explanation of the clinical relevance of chosen efficacy and harm outcomes is strongly recommended	13<br><br>Lines 246-267	

Participant timeline	#13	Time schedule of enrolment, interventions (including any run-ins and washouts), assessments, and visits for participants. A schematic diagram is highly recommended (see Figure)	Table 1<br><br>Page 27-28, lines 556-563	
Sample size	#14	Estimated number of participants needed to achieve study objectives and how it was determined, including clinical and statistical assumptions supporting any sample size calculations	14-15<br><br>Lines 284-291	
Recruitment	#15	Strategies for achieving adequate participant enrolment to reach target sample size	18<br><br>Lines 360-363	
**Methods: Assignment of interventions (for controlled trials)**				
Allocation: sequence generation	#16a	Method of generating the allocation sequence (eg, computer-generated random numbers), and list of any factors for stratification. To reduce predictability of a random sequence, details of any planned restriction (eg, blocking) should be provided in a separate document that is unavailable to those who enrol participants or assign interventions	9<br><br>Lines 147-149	
Allocation concealment mechanism	#16b	Mechanism of implementing the allocation sequence (eg, central telephone; sequentially numbered, opaque, sealed	9<br><br>Lines 145-155	

		envelopes), describing any steps to conceal the sequence until interventions are assigned		
Allocation: implementation	#16c	Who will generate the allocation sequence, who will enrol participants, and who will assign participants to interventions	9<br><br>Lines 145-155	
Blinding (masking)	#17a	Who will be blinded after assignment to interventions (eg, trial participants, care providers, outcome assessors, data analysts), and how	9<br><br>Lines 149-152	
Blinding (masking): emergency unblinding	#17b	If blinded, circumstances under which unblinding is permissible, and procedure for revealing a participant's allocated intervention during the trial	9<br><br>Lines 149-152	
**Methods: Data collection, management, and analysis**				
Data collection plan	#18a	Plans for assessment and collection of outcome, baseline, and other trial data, including any related processes to promote data quality (eg, duplicate measurements, training of assessors) and a description of study instruments (eg, questionnaires, laboratory tests) along with their reliability and validity, if known. Reference to where data collection forms can be found, if not in the protocol	18-19<br><br>Lines 377-389	
Data collection plan: retention	#18b	Plans to promote participant retention and complete follow-up, including list of any outcome data to be collected for participants	Page 10, Lines 174-181<br><br>Page 10, Lines 188-189	

		who discontinue or deviate from intervention protocols	Page 11, Lines 211-213	
Data management	#19	Plans for data entry, coding, security, and storage, including any related processes to promote data quality (eg, double data entry; range checks for data values). Reference to where details of data management procedures can be found, if not in the protocol	18-19<br><br>Lines 377-389	
Statistics: outcomes	#20a	Statistical methods for analysing primary and secondary outcomes. Reference to where other details of the statistical analysis plan can be found, if not in the protocol	16<br><br>Lines 311-332	
Statistics: additional analyses	#20b	Methods for any additional analyses (eg, subgroup and adjusted analyses)	15<br><br>Lines 301-309	
Statistics: analysis population and missing data	#20c	Definition of analysis population relating to protocol non-adherence (eg, as randomised analysis), and any statistical methods to handle missing data (eg, multiple imputation)	15<br><br>Lines 301-309	Statistical methods to handle missing data can be found in the Statistical Analysis Plan, which is still in development. Details regarding access to the statistical analysis plan (SAP) will be provided alongside the primary manuscript, which will present the study findings.
**Methods: Monitoring**				
Data monitoring: formal committee	#21a	Composition of data monitoring committee (DMC); summary of its role and reporting	19<br><br>Lines 390-393	

		structure; statement of whether it is independent from the sponsor and competing interests; and reference to where further details about its charter can be found, if not in the protocol. Alternatively, an explanation of why a DMC is not needed		
Data monitoring: interim analysis	#21b	Description of any interim analyses and stopping guidelines, including who will have access to these interim results and make the final decision to terminate the trial	15<br><br>Lines 293-299	
Harms	#22	Plans for collecting, assessing, reporting, and managing solicited and spontaneously reported adverse events and other unintended effects of trial interventions or trial conduct	14<br><br>Lines 269-281	
Auditing	#23	Frequency and procedures for auditing trial conduct, if any, and whether the process will be independent from investigators and the sponsor	19<br><br>Lines 394-399	
**Ethics and dissemination**				
Research ethics approval	#24	Plans for seeking research ethics committee / institutional review board (REC / IRB) approval	23<br><br>Lines 457-459	
Protocol amendments	#25	Plans for communicating important protocol modifications (eg, changes to eligibility criteria, outcomes, analyses) to relevant parties (eg, investigators, REC / IRBs, trial	17<br><br>Lines 348-351	

		participants, trial registries, journals, regulators)		
Consent or assent	#26a	Who will obtain informed consent or assent from potential trial participants or authorised surrogates, and how (see Item 32)	17-18<br><br>Lines 352-359	
Consent or assent: ancillary studies	#26b	Additional consent provisions for collection and use of participant data and biological specimens in ancillary studies, if applicable	Page 18; Lines 370-375<br><br>Pages 19-20; Lines 402 -405	
Confidentiality	#27	How personal information about potential and enrolled participants will be collected, shared, and maintained in order to protect confidentiality before, during, and after the trial	18-19<br><br>Lines 377-389	
Declaration of interests	#28	Financial and other competing interests for principal investigators for the overall trial and each study site	24<br><br>Lines 473-478	
Data access	#29	Statement of who will have access to the final trial dataset, and disclosure of contractual agreements that limit such access for investigators	23<br><br>Lines 465-470	
Ancillary and post trial care	#30	Provisions, if any, for ancillary and post-trial care, and for compensation to those who suffer harm from trial participation	20<br><br>Lines 407-408	
Dissemination policy: trial results	#31a	Plans for investigators and sponsor to communicate trial results to participants,	20<br><br>Lines 410-417	

		healthcare professionals, the public, and other relevant groups (eg, via publication, reporting in results databases, or other data sharing arrangements), including any publication restrictions		
Dissemination policy: authorship	#31b	Authorship eligibility guidelines and any intended use of professional writers	20<br><br>Lines 410-417	
Dissemination policy: reproducible research	#31c	Plans, if any, for granting public access to the full protocol, participant-level dataset, and statistical code	23<br><br>Lines 465-470	
**Appendices**				
Informed consent materials	#32	Model consent form and other related documentation given to participants and authorised surrogates	23<br><br>Lines 462-463	We provided the model prescreening and main consent forms for the trial. These files have been uploaded as supplemental files not for publication
Biological specimens	#33	Plans for collection, laboratory evaluation, and storage of biological specimens for genetic or molecular analysis in the current trial and for future use in ancillary studies, if applicable	Page 18; Lines 370-375<br><br>Pages19-20; Lines 402-405	

It is strongly recommended that this checklist be read in conjunction with the SPIRIT 2013 Explanation & Elaboration for important clarification on the items. Amendments to the protocol should be tracked and dated. The SPIRIT checklist is copyrighted by the SPIRIT Group under the Creative Commons "Attribution-NonCommercial-NoDerivs 3.0 Unported" license. This checklist can be completed online using https://www.goodreports.org/, a tool made by the EQUATOR Network in collaboration with Penelope.ai
